# Bloodstream infections after solid organ transplantation: clinical epidemiology and antimicrobial resistance (2016–21)

**DOI:** 10.1093/jacamr/dlad158

**Published:** 2024-01-11

**Authors:** Max W Adelman, Ashton A Connor, Enshuo Hsu, Ashish Saharia, Constance M Mobley, David W Victor, Mark J Hobeika, Jiejian Lin, Kevin A Grimes, Elizabeth Ramos, Claudia Pedroza, Elizabeth W Brombosz, R Mark Ghobrial, Cesar A Arias

**Affiliations:** Division of Infectious Diseases, Department of Medicine, Houston Methodist Hospital, Houston, TX, USA; Center for Infectious Diseases, Houston Methodist Research Institute, Houston, TX, USA; Division of Pulmonary, Critical Care, and Sleep Medicine, Houston Methodist Hospital, Houston, TX, USA; Department of Medicine, Weill Cornell Medical College, NewYork, NY, USA; Department of Surgery, Weill Cornell Medical College, New York, NY, USA; Department of Surgery, Houston Methodist Hospital, Houston, TX, USA; J.C. Walter, Jr. Transplant Center, Houston Methodist Hospital, Houston, TX, USA; Center for Health Data Science and Analytics, Houston Methodist Hospital, Houston, TX, USA; Department of Surgery, Weill Cornell Medical College, New York, NY, USA; Department of Surgery, Houston Methodist Hospital, Houston, TX, USA; J.C. Walter, Jr. Transplant Center, Houston Methodist Hospital, Houston, TX, USA; Department of Surgery, Weill Cornell Medical College, New York, NY, USA; Department of Surgery, Houston Methodist Hospital, Houston, TX, USA; J.C. Walter, Jr. Transplant Center, Houston Methodist Hospital, Houston, TX, USA; J.C. Walter, Jr. Transplant Center, Houston Methodist Hospital, Houston, TX, USA; Department of Surgery, Weill Cornell Medical College, New York, NY, USA; Department of Surgery, Houston Methodist Hospital, Houston, TX, USA; J.C. Walter, Jr. Transplant Center, Houston Methodist Hospital, Houston, TX, USA; Division of Infectious Diseases, Department of Medicine, Houston Methodist Hospital, Houston, TX, USA; Department of Medicine, Weill Cornell Medical College, NewYork, NY, USA; Division of Infectious Diseases, Department of Medicine, Houston Methodist Hospital, Houston, TX, USA; Center for Infectious Diseases, Houston Methodist Research Institute, Houston, TX, USA; Department of Medicine, Weill Cornell Medical College, NewYork, NY, USA; Division of Infectious Diseases, Department of Medicine, Houston Methodist Hospital, Houston, TX, USA; Center for Clinical Research and Evidence-Based Medicine, The University of Texas Health Science Center at Houston, Houston, TX, USA; Department of Surgery, Houston Methodist Hospital, Houston, TX, USA; Department of Surgery, Weill Cornell Medical College, New York, NY, USA; Department of Surgery, Houston Methodist Hospital, Houston, TX, USA; J.C. Walter, Jr. Transplant Center, Houston Methodist Hospital, Houston, TX, USA; Division of Infectious Diseases, Department of Medicine, Houston Methodist Hospital, Houston, TX, USA; Center for Infectious Diseases, Houston Methodist Research Institute, Houston, TX, USA; Department of Medicine, Weill Cornell Medical College, NewYork, NY, USA

## Abstract

**Background:**

Solid organ transplant (SOT) recipients are at risk of bloodstream infections (BSIs) with MDR organisms (MDROs).

**Objectives:**

To describe the epidemiology of BSI in the year after several types of SOT, as well as the prevalence of MDRO infections in this population.

**Methods:**

We conducted a single-centre, retrospective study of kidney, liver, heart, and multi-organ transplantation patients. We examined BSIs ≤1 year from SOT and classified MDRO phenotypes for *Staphylococcus aureus*, enterococci, Enterobacterales, *Pseudomonas aeruginosa* and *Candida* spp. We compared BSI characteristics between SOT types and determined risk factors for 90 day mortality.

**Results:**

We included 2293 patients [1251 (54.6%) kidney, 663 (28.9%) liver, 219 (9.6%) heart and 160 (7.0%) multi-organ transplant]. Overall, 8.5% of patients developed a BSI. BSIs were most common after multi-organ (23.1%) and liver (11.3%) transplantation (*P* < 0.001). Among 196 patients with BSI, 323 unique isolates were recovered, 147 (45.5%) of which were MDROs. MDROs were most common after liver transplant (53.4%). The most frequent MDROs were VRE (69.8% of enterococci) and ESBL-producing and carbapenem-resistant Enterobacterales (29.2% and 27.2% of Enterobacterales, respectively). Mortality after BSI was 9.7%; VRE was independently associated with mortality (adjusted OR 6.0, 95% CI 1.7–21.3).

**Conclusions:**

BSI incidence after SOT was 8.5%, with a high proportion of MDROs (45.5%), especially after liver transplantation. These data, in conjunction with local antimicrobial resistance patterns and prescribing practices, may help guide empirical antimicrobial selection and stewardship practices after SOT.

## Introduction

Solid organ transplantation (SOT) is a lifesaving procedure for patients with organ failure, and nearly 40 000 organ transplants are performed yearly in the USA.^1,2^ Although SOT improves patient survival and quality of life,^2,3^ patients are at high risk of post-transplantation complications, including bloodstream infection (BSI).^4^ For example, BSIs occur in up to 30% of patients after liver transplantation,^4–7^ and infections contribute to a significant proportion of deaths in this population.^8^

While all SOT patients are at increased risk of BSI, BSI risk varies between SOT types due to multiple factors including intensity of immunosuppression, direct intestinal disruption during surgery, and likelihood of post-operative critical illness.^4,9^ Further, operative and anatomical factors contribute to infections with specific types of bacteria.^10^ For example, liver transplant recipients are at high risk of BSI with enterococci, frequently because of biliary complications and intestinal domination,^4,7,11^ whereas kidney transplant recipients are at higher risk of BSI due to urinary tract pathogens such as *Escherichia coli* and *Klebsiella* spp.^10,12^

Over the past several years, the frequency of infections due to several MDR organisms (MDROs), including ESBL-producing Enterobacterales and VRE, has increased among hospitalized patients.^13^ Infection with an MDRO increases mortality,^14–17^ and transplant patients are at high risk of developing MDRO infections due to multiple factors, including frequent use of antibiotics after organ transplantation.^17^

Despite the increasing burden of MDROs and their impact on outcomes among transplant patients, there have been few recent data from the USA characterizing the clinical epidemiology of MDROs among SOT recipients. Here, using a comprehensive database of transplant patients (2016–21), we characterized the frequency of BSI after different types of SOT and assessed the rates of BSI due to MDROs and their association with mortality.

## Patients and methods

### Patients and data collection

This study was approved by the Houston Methodist Research Institute Institutional Review Board (Protocol #00000587) with a waiver of informed consent. This was a single-centre, retrospective study of patients ≥18 years old who underwent kidney, liver, heart or multi-organ transplantation (i.e. heart/liver, heart/kidney, liver/kidney or heart/liver/kidney) during the same hospitalization from 1 June 2016 through to 30 September 2021. We collected clinical data through to 1 January 2023 to allow for determination of BSI in the year after SOT (latest possible BSI onset date: 30 September 2022), as well as 3 month mortality after BSI (latest possible death date: 1 January 2023). We excluded patients transplanted with an organ other than kidney, liver or heart, patients who underwent dual-organ transplantation during separate hospitalizations, and patients who had re-transplant of the same organ. We extracted clinical and demographic variables from the electronic medical record (Epic, Epic Systems, WI, USA), including ICD-10 codes, which were used to determine the Charlson comorbidity index.^18^

### Microbiological analyses

We evaluated blood cultures drawn for clinical care in the first year after SOT. Isolate identification was performed by the microbiology laboratory using MALDI-TOF (Bruker, MA, USA) and antimicrobial susceptibility determined with BD Phoenix (BD, NJ, USA). We did not count common commensals/contaminants (as defined by the CDC) as causing a BSI unless the same organism was isolated within 3 calendar days of the index culture, per CDC guidelines.^19,20^ If the same non-commensal organism was isolated from a blood culture within 14 days of an index positive culture, this organism was not counted, based on CDC guidelines. If the same organism was isolated >14 days from an index positive culture, this isolate was counted as a separate BSI episode, and both the episode and isolate were analysed separately from the first episode.^21^ If two different organisms were isolated from the blood within 3 calendar days of each other, this was considered a single BSI episode, but organism characteristics (e.g. species and MDRO type) were analysed separately. We analysed both the total number of BSI episodes as well as total number of unique isolates.

We focused the analysis on five groups of organisms: *Staphylococcus aureus*, *Enterococcus* spp., Enterobacterales, *Pseudomonas aeruginosa* and *Candida* spp. Antimicrobial susceptibilities for specific isolate–antimicrobial combinations were determined using CLSI guidelines as applied by the clinical microbiology laboratory. The definition of MDRO in each organism group was based on CDC definitions^22^ (Table S1, available as Supplementary data at *JAC-AMR* Online) and included MRSA, VRE, ESBL-producing Enterobacterales, carbapenem-resistant Enterobacterales (CRE), carbapenem non-susceptible *P. aeruginosa* (CNSPA), and azole-resistant *Candida* (ARC) spp. There was no routine screening performed for any of these organisms during this study.

### Statistical methods

We compared demographics, clinical information and BSI characteristics between kidney, liver, heart and multi-organ transplant recipients. Continuous variables were compared with ANOVA and categorical variables with *χ*^2^ or Fisher’s exact tests as appropriate. We examined 90 day mortality after BSI onset and considered only the first BSI in mortality analyses for patients with multiple BSI episodes. We performed univariable and multivariable logistic regression to determine whether specific BSI types (i.e. organisms and MDRO types) were associated with 90 day mortality. Covariates for the multivariable models were selected based on clinical importance and potential for confounding the association between BSI type and mortality. We constructed separate multivariable models for each organism type and MDRO type (i.e. the referent was all other BSIs). *P* values of ≤0.05 were considered statistically significant. Analyses were performed in SAS OnDemand (SAS Institute, NC, USA), Excel (Microsoft, WA, USA), R [R version 4.2.2 (2022-10-31)] and R Studio (version 2022.12.0 + 353) using the Tidyverse, table1, ggplot2 and RColorBrewer packages.

## Results

### Patient characteristics

**Figure 1.**
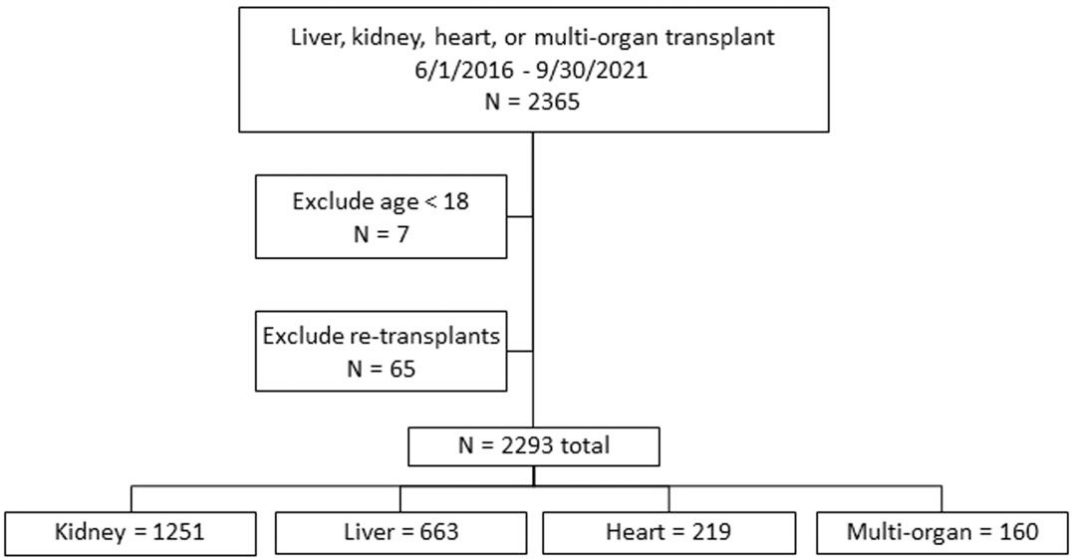
Flow diagram of SOT patients included.

Overall, 2293 transplant patients were included (Figure 1): 1251 (54.6%) kidney, 663 (28.9%) liver, 219 (9.6%) heart and 160 (7.0%) multi-organ transplant recipients. Of the multi-organ recipients, 85 received kidney/liver, 50 heart/kidney, 22 heart/liver and 3 heart/kidney/liver transplants. Baseline demographics at time of transplant are shown in Table 1. Mean age of the cohort was 53.4 years (SD ± 13.0), 909 (39.6%) were female, 1547 (67.5%) were white and 542 (23.6%) were Hispanic/Latino. Overall, 457 patients (19.9%) were in the ICU immediately prior to transplant, and the proportion of ICU patients was highest among heart (65.8%) and multi-organ transplant patients (53.1%, overall *P* < 0.001). Mean Charlson comorbidity index was 5.6 (SD ± 3.6) and was highest among multi-organ (9.0, SD ± 3.7) and liver transplant recipients (7.4, SD ± 3.8) (*P* < 0.001) (Table 1). Standard institutional maintenance immunosuppression and antimicrobial prophylaxis regimens are listed in Tables S2 and S3, respectively.

**Table 1.** Characteristics of SOT patients included

	SOT type				
Characteristic	Overall (*n* = 2293)	Kidney (*n* = 1251)	Liver (*n* = 663)	Heart (*n* = 219)	Multi-organ (*n* = 160)
Age, years, mean (SD)	53.4 (13.0)	51.0 (13.3)	56.1 (11.8)	55.3 (12.7)	57.8 (11.3)
Female, *n* (%)	909 (39.6)	533 (42.6)	263 (39.7)	55 (25.1)	58 (36.3)
Race, *n* (%)					
Asian	154 (6.7)	115 (9.2)	28 (4.2)	5 (2.3)	6 (3.8)
Black	500 (21.8)	347 (27.7)	47 (7.1)	62 (28.3)	44 (27.5)
Unknown	63 (2.7)	39 (3.1)	14 (2.1)	6 (2.7)	4 (2.5)
White	1547 (67.5)	729 (58.3)	567 (85.5)	146 (66.7)	105 (65.6)
Other	29 (1.3)	21 (1.7)	7 (1.1)	0 (0)	1 (0.6)
Hispanic/Latino, *n* (%)	542 (23.6)	325 (26.0)	148 (22.3)	28 (12.8)	41 (25.6)
BMI, kg/m^2^, mean (SD)	27.8 (5.57)	27.7 (5.09)	28.6 (6.43)	26.6 (4.39)	26.1 (5.86)
In ICU at time of transplant, *n* (%)	457 (19.9)	0 (0)	228 (34.4)	144 (65.8)	85 (53.1)
CCI, mean (SD)	5.6 (3.6)	4.2 (2.7)	7.4 (3.8)	5.7 (3.1)	9.0 (3.7)
MI, *n* (%)	317 (13.8)	121 (9.7)	43 (6.5)	98 (44.7)	55 (34.4)
CHF, *n* (%)	632 (27.6)	236 (18.9)	97 (14.6)	195 (89.0)	104 (65.0)
PVD, *n* (%)	1415 (61.7)	812 (64.9)	494 (74.5)	42 (19.2)	67 (41.9)
CVD, *n* (%)	717 (31.3)	193 (15.4)	295 (44.5)	132 (60.3)	97 (60.6)
Pulmonary disease, *n* (%)	367 (16.0)	123 (9.8)	144 (21.7)	62 (28.3)	38 (23.8)
Mild liver disease, *n* (%)	899 (39.2)	120 (9.6)	622 (93.8)	43 (19.6)	114 (71.3)
Mod/severe liver disease, *n* (%)	691 (30.1)	5 (0.4)	579 (87.3)	10 (4.6)	97 (60.6)
DM w/o complications, *n* (%)	737 (32.1)	366 (29.3)	219 (33.0)	72 (32.9)	80 (50.0)
DM w/complications, *n* (%)	535 (23.3)	333 (26.6)	92 (13.9)	48 (21.9)	62 (38.8)
Renal disease, *n* (%)	1659 (72.4)	1175 (93.9)	210 (31.7)	127 (58.0)	147 (91.9)
Cancer, *n* (%)	374 (16.3)	73 (5.8)	254 (38.3)	19 (8.7)	28 (17.5)
Metastatic cancer, *n* (%)	108 (4.7)	32 (2.6)	61 (9.2)	4 (1.8)	11 (6.9)
HIV/AIDS, *n* (%)	19 (0.8)	14 (1.1)	5 (0.8)	0 (0)	0 (0)

CCI, Charlson comorbidity index; CHF, congestive heart failure; CTD, connective tissue disease; CVD, cerebrovascular disease; DM, diabetes mellitus; MI, myocardial infarction; PUD, peptic ulcer disease; PVD, peripheral vascular disease.

### BSIs

**Figure 2.**
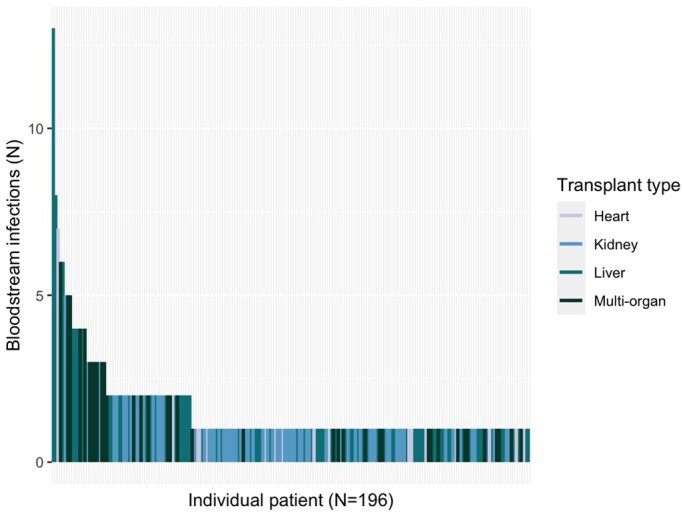
Count of BSI episodes per individual patient, colour-coded by organ transplant type. Heart and kidney transplant recipients (lighter colours) had fewer BSI episodes, whereas liver and multi-organ transplant recipients (darker colours) were more highly represented among patients with multiple BSIs.

Overall, 8.5% of the cohort (*n* = 196) developed at least one BSI in the year after transplant. The frequency was highest in multi-organ transplant recipients (23.1%), followed by liver (11.3%), heart (7.8%) and kidney transplant recipients (5.4%) (*P* < 0.001). Among these 196 patients, there were 313 BSI episodes, 10 of which (3.5%) included two organisms, yielding a total of 323 unique isolates. Of note, 29.1% of patients with a BSI (*n* = 57) had multiple BSI episodes; multiple BSIs were most common in multi-organ (48.7%) and liver transplant (29.3%) recipients, with a maximum number of episodes of 6 and 13, respectively (*P* < 0.001 compared with other organ types) (Figure 2). A total of 34 (17.3%) patients who had a BSI had additional BSIs with the same organism (i.e. the same species collected >14 days from index positive culture). Of note, 79 BSIs (25.2%) had ICU onset and ICU-onset BSIs were most common among liver transplant patients (38.9% of liver transplant patients with BSI, *P* < 0.001 compared with other organ types).

The most common organisms isolated were *Klebsiella* spp., which accounted for 21.1% (*n* = 68) of 323 isolates, followed by *E. coli* (19.8%; *n* = 64), *Enterococcus faecium* (15.8%; *n* = 51), *P. aeruginosa* (8.4%; *n* = 27) and *S. aureus* (6.5%; *n* = 21) (Table S4). When organisms were clustered by groups of interest (see Patients and methods), the most common were Enterobacterales (48.0%; *n* = 155), followed by *Enterococcus* spp. (20.7%, *n* = 67), *P. aeruginosa* (8.4%, *n* = 27), *S. aureus* (6.5%, *n* = 21) and *Candida* spp. (5.0%, *n* = 16) (Table 2). Enterobacterales were most prevalent among kidney transplant recipients (60.9% of BSIs in this group, *P* = 0.002), although >35% of BSIs among each organ type were caused by Enterobacterales. In contrast, *Enterococcus* spp. were the most prevalent among liver transplant patients (32.3%, *P* < 0.001) and were relatively rare after kidney transplantation (3.4%). *P. aeruginosa* and *S. aureus* were numerically most common after heart (19.2%) and kidney transplant (12.6%), respectively, although differences in these organisms were not statistically significant by organ type.

**Table 2.** Organism groups and associated antimicrobial resistance causing BSIs in the year after SOT

	SOT type					
Organism	Overall (*n* = 323)	Kidney (*n* = 87)	Liver (*n* = 133)	Heart (*n* = 26)	Multi-organ (*n* = 77)	*P* ^a^
Enterobacterales	155 (48.0)	53 (60.9)	48 (36.1)	12 (46.2)	42 (54.5)	0.002
ESBL (% of all organisms)	52 (16.1)	23 (26.4)	14 (10.5)	3 (11.5)	12 (15.6)	0.02
ESBL (% of Enterobacterales)	52 (33.6)	23 (43.4)	14 (29.2)	3 (25.0)	12 (28.6)	0.31
CRE (% of all organisms)	20 (6.2)	0 (0)	13 (9.8)	1 (3.9)	6 (7.8)	0.03
CRE (% of Enterobacterales)	20 (12.9)	0 (0)	13 (27.1)	1 (8.3)	6 (14.3)	<0.001
*Enterococcus* spp.	67 (20.7)	3 (3.4)	43 (32.3)	7 (26.9)	14 (18.2)	<0.001
VRE (% of all organisms)	45 (13.9)	0 (0)	30 (22.6)	3 (11.5)	12 (15.6)	<0.001
VRE (% of *Enterococcus*)	45 (67.2)	0 (0)	30 (69.8)	3 (42.9)	12 (85.7)	0.02
*P. aeruginosa*	27 (8.4)	7 (8.0)	13 (9.8)	5 (19.2)	2 (2.6)	0.05
CNSPA (% of all organisms)	12 (3.7)	1 (1.1)	8 (6.0)	1 (3.9)	2 (2.6)	0.28
CNSPA (% of *Pseudomonas*)	12 (44.4)	1 (14.3)	8 (61.5)	1 (20)	2 (100)	0.05
*S. aureus*	21 (6.5)	11 (12.6)	6 (4.5)	1 (3.8)	3 (3.9)	0.06
MRSA (% of all organisms)	8 (2.5)	4 (4.6)	2 (1.5)	0 (0)	2 (2.6)	0.42
MRSA (% of *S. aureus*)	8 (38.1)	4 (36.4)	2 (33.3)	0 (0)	2 (66.7)	0.63
*Candida* spp.	16 (5.0)	3 (3.4)	6 (4.5)	0 (0)	7 (9.1)	0.20
ARC (% of all organisms)	10 (3.1)	2 (2.3)	4 (3.0)	0 (0)	4 (5.2)	0.54
ARC (% of *Candida*)	10 (62.5)	2 (66.7)	4 (66.7)	0 (0)	4 (57.1)	0.93
Any MDRO^b^	147 (45.5)	30 (34.5)	71 (53.4)	8 (30.8)	38 (49.4)	0.02
Other organisms	37 (11.5)	10 (11.5)	17 (12.8)	1 (3.8)	9 (11.7)	0.63

^a^ *P* value (determined by *χ*^2^ or ANOVA) is comparison between different organ transplant types.

^b^Any organism that was either ESBL-producing (ESBL), CRE, VRE, CNSPA, MRSA or ARC was counted as an MDRO.

**Figure 3.**
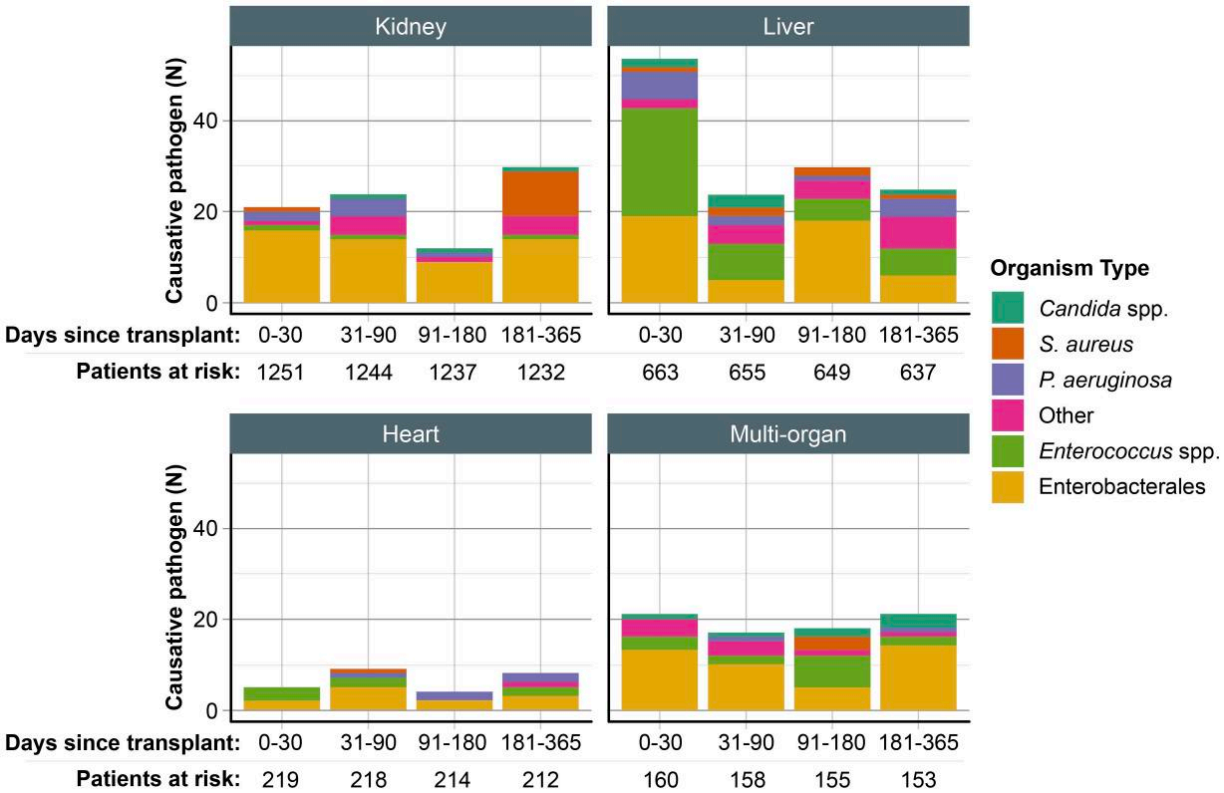
Number of BSIs caused by different organisms, grouped by organ transplant type and time since transplant.

Across all transplant types, 31.3% BSIs occurred in the first 30 days after transplant, and most BSIs (54.2%) occurred in the first 90 days after transplant (Figure S1). Across all time periods, Enterobacterales were the most common organisms isolated (49.5% from 0 to 30 days, 46.0% from 31 to 90 days, 53.1% from 91 to 180 days and 44.1% from 181 to 365 days). *Enterococcus* spp. were more common in the first 30 days after transplant (30.7%) than in the other time periods (17.6%, 18.8% and 13.1%, respectively), and this feature was driven by a large number of *Enterococcus* spp. in the first 30 days after liver transplantation [44.4% BSI isolates (*n* = 24) in this time period among liver transplant patients] (Figure 3). Among SOT types, the highest proportion of BSIs in the first 30 days after transplant occurred among liver transplant patients (40.6%), whereas the proportion of BSIs across time periods were relatively stable for other organ types (noting that the time periods differ in length).

### Antimicrobial resistance patterns

**Figure 4.**
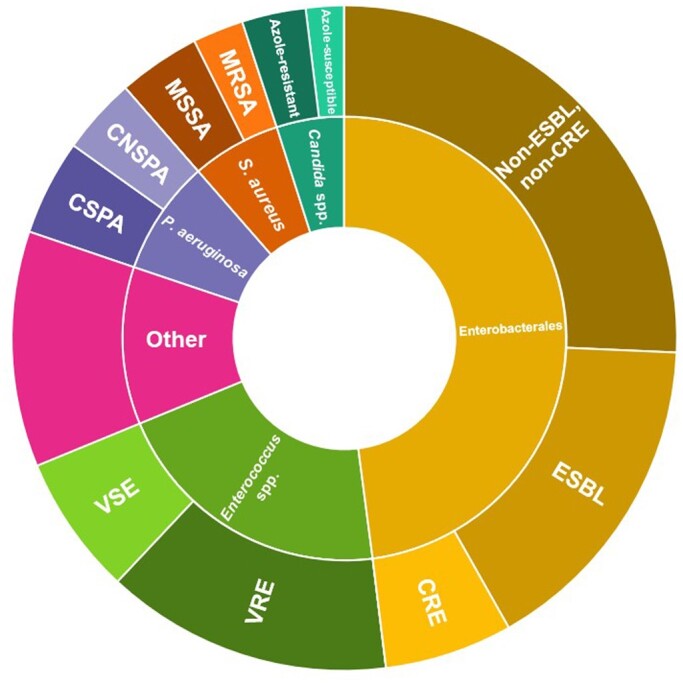
Isolates causing BSIs (*n* = 323), categorized by antimicrobial resistance profiles, in the year after SOT. Areas are proportional to number of BSIs due to each specific organism. CSPA, carbapenem-susceptible *P. aeruginosa*; VSE, vancomycin-susceptible *Enterococcus*.

The proportions of MDRO isolates are shown in Table 2 and Figure 4. Overall, a high proportion of organisms were MDROs (as defined in Table S1). Out of 323 isolates, 147 (45.5%) were MDROs (either MRSA, VRE, ESBL producers, CRE, CNSPA or ARC). The proportion of MDROs varied across organ transplant types. Liver transplant recipients harboured the most MDROs in BSIs (53.4% of all isolates), followed by multi-organ transplant recipients (49.4%, *P* = 0.02 compared with other organ types). Of 155 Enterobacterales isolates, 33.6% (*n* = 52) were ESBL producers and 12.9% (*n* = 20) were CRE. Of note, among kidney transplant recipients, 43% of Enterobacterales isolates were ESBL producers (*n* = 23) but there were no CRE. In contrast, CRE were most common among liver transplant recipients (27.1% of all Enterobacterales, *P* < 0.001 compared with other organ transplant types).

Most enterococci (76.1%, *n* = 51) were *E. faecium*, and 67.2% of all enterococci (*n* = 45) were VRE. Among enterococci causing BSIs in multi-organ and liver transplant patients, 85.7% and 69.8% were VRE, respectively, and these organ types were most likely to have VRE BSI (*P* < 0.02 compared with other organ transplant types). Of note, 62.5% of *Candida* spp. isolates were ARC, driven by a large proportion of *Candida glabrata*. Furthermore, among *P. aeruginosa* BSI, 44.4% were CNSPA and 18.5% were MDR according to CDC criteria.^22^

### Outcomes

Overall 90 day mortality after BSI onset was 9.7% (19/196 patients with BSI). Comparing SOT types, mortality was numerically highest among liver transplant recipients (14.7%, 11/75 patients with BSI), but this was not statistically significant (*P* = 0.26). On univariable and multivariable analysis, no specific patient characteristic (including age, race, gender, transplant type, ICU admission at time of transplant, and Charlson comorbidity index) was associated with mortality after BSI (Table S5). Among organism groups, patients with Enterobacterales BSI had lower 90 day mortality on univariable analysis compared with all other BSI types (OR 0.3, 95% CI 0.1–1.0); however, this was not significant after controlling for patient characteristics listed above (adjusted OR 0.4, 95% CI 0.1–1.3). The only patient or organism characteristic independently associated with 90 day mortality after BSI was VRE (adjusted OR 6.0, 95% CI 1.7–21.3), which was significantly associated with mortality in both univariable and multivariable models. Compared with patients with non-MDRO BSI, patients with BSI due to any MDRO did not have higher mortality (OR 1.5, 95% CI 0.6–3.9).

## Discussion

In this large, single-centre retrospective cohort study of BSI in the first year after SOT, we found that BSI incidence was 8.5% and differed significantly by SOT type, with multi-organ (23.1%) and liver (11.3%) transplant recipients at highest risk. A substantial proportion (45.5%) of isolates were MDROs, including relatively high proportions of ESBL-producing Enterobacterales (16.1% of all organisms), VRE (13.9%) and CRE (6.2%). Liver transplant recipients were at highest risk for MDRO BSI (53.4% of BSIs among liver transplant recipients), including with VRE (22.6%) and CRE (9.8%). VRE was independently associated with increased mortality among patients with BSI (adjusted OR 6.0).

Despite the impact of BSIs on post-SOT mortality,^4,8,23^ there have been few recent US studies examining the landscape of BSIs, including BSIs due to MDROs, after SOT. Further, comparisons between existing studies are limited due to varying timeframes and geography, as well as specific organ types examined. Our study found a lower incidence of BSI (8.5%) in the first year after SOT than a recent study utilizing a national registry from Denmark (13.3% overall), as well as lower incidence among liver (11.3% versus 17.7%) and kidney (5.4% versus 11.8%), although higher incidence among heart transplant recipients (7.8% versus 2.2%).^4^ Incidence in our study was very similar to a recent report from Switzerland, which found overall incidence of 9.5%, including 11.4% among liver transplant recipients.^24^ Although the Danish study used a relatively contemporaneous time period (2010–17), we report consistently lower incidence of BSI than older studies that focused on liver^5,6,25–27^ and kidney transplant recipients.^17^ Some of this variation may reflect geographical differences, although improved infection control practices, as well as the experience of our large-volume transplant centre, may explain the apparent improvement in trends over time.

Despite a relatively low number of BSIs in our cohort, a substantial proportion of BSI organisms (45.5%) were MDROs. We chose to examine specific MDROs (MRSA, VRE, ESBL producers, CRE, CNSPA and ARC) that are clinically relevant and most likely to impact empirical and definitive antimicrobial selection. We found that liver transplant patients were at highest risk of MDRO BSI, which may be related to high rates of ICU utilization and antimicrobial exposures in this population.^28,29^ CRE specifically made up 6.2% of all isolates and 12.9% of Enterobacterales, similar to proportions reported in a recent multicentre study.^30^ The fact that kidney transplant recipients had a higher proportion of Enterobacterales (60.9%) than liver transplant recipients (36.1%) but no CRE may further reflect the selective pressure from peri-operative critical illness (and associated antibiotic exposure) among liver transplant patients. Interestingly, although CRE infection has been linked to high mortality,^30,31^ there were no deaths among patients with CRE BSIs in our cohort. Whether novel β-lactam/β-lactamase inhibitor combinations, which were introduced during our study period, may have contributed to this low mortality deserves further study. Indeed, these agents have improved toxicity profiles and appear to decrease mortality compared with traditional regimens in small retrospective cohort studies.^32^

Among patients with BSI, the only MDRO that was associated with 90 day mortality was VRE, which independently increased the odds of death 6-fold compared with patients with non-VRE BSI. VRE were highly prevalent among liver transplant patients, where they made up more than 20% of all organisms and nearly 70% of enterococci. There are scant data specifically on enterococci and VRE infections after liver transplant or SOT in general. Two studies published in 1999 and 2006 reported VRE colonization between 13% and 18% after liver transplantation^33,34^ and 1 year mortality after VRE infection as high as 82%.^33^ Although contemporary VRE-related mortality is likely much lower, it remains unacceptably high despite introduction of bactericidal antibiotics with relatively safe profiles such as daptomycin. Clearly, research to mitigate morbidity and mortality from enterococcal BSI is urgently needed, including study of non-antibiotic therapies such as microbiome manipulation for VRE colonization eradication.

There are several other interesting findings that deserve to be discussed. First, a high proportion of patients, especially liver and multi-organ transplant recipients, developed multiple BSI episodes. Recurrent BSI after SOT has been noted in prior studies,^5,7,25^ but has not been specifically studied outside of one paper that showed that recurrent BSI was associated with increased mortality.^25^ Prospective translational cohort studies are needed to identify potential reservoirs of recurrent BSI in these high-risk patients, and to determine whether recurrent BSI generally reflects inadequate source control, antibiotic failure due to antibiotic resistance, or other factors. Second, we were surprised at the relatively low proportion of *Candida* BSIs in our cohort (5% of all isolates), including among high-risk groups such as liver (4.5% of all isolates) and multi-organ transplant recipients (9.1%). This is lower than ICU-onset *Candida* BSI reported in a recent national cohort study.^35^ The low rate of *Candida* BSI, as well as relatively high proportion of ARC, may be driven by antifungal prophylaxis at our centre;^36^ prospective studies on antifungal prophylaxis are warranted. Lastly, our institution is a high-volume centre nationally for several multi-organ transplants, including heart/liver and liver/kidney. The finding that multi-organ transplant recipients have a significant BSI burden (23.1%) deserves further study since this characteristic has not previously been shown.

Our study has several limitations. First, it is a single-centre study, which limits generalizability of our findings. We view this study as hypothesis-generating and our findings should be interpreted in the context of local epidemiology and risk of MDROs. Second, we did not include data on the impact of several potential confounders (e.g. antimicrobials, immunosuppression and surgical complications) on BSI and MDRO risk. While important, these topics have been addressed by excellent contemporary studies^28,30^ and were outside the scope of our study, which aimed to describe the landscape of BSI and MDROs after several types of organ transplantation. Third, we used arbitrary cut-offs (although recommended by the CDC) to define when a positive blood culture would count as a new episode versus continuation of the index episode.^21^ We did not collect isolates to allow for WGS, which would have increased our ability to determine whether subsequent isolates represented the same or different BSI episodes. Regardless, the high rate of recurrent and multiple BSI episodes is alarming. Fourth, we excluded patients with re-transplantation, which may have led to an underestimation of BSI incidence, especially among liver transplant patients (who may require re-transplant due to conditions associated with infection, including ischaemic cholangiopathy). Lastly, there are likely unmeasured confounders that we did not account for in our multivariable models. Additionally, ICD-10 codes may not accurately reflect comorbidities. These are both common challenges in retrospective cohort studies.

In summary, we demonstrated BSI incidence of 8.5% and an associated high MDRO burden among SOT patients in a high-volume transplant centre. Although BSI incidence was lower than in previous studies, the emergence of MDR phenotypes, especially CRE and VRE infections after liver and multi-organ transplantation, is worrisome. Our results can guide future research on strategies to decrease burden and impact of MDROs after SOT.

## Supplementary Material

dlad158_Supplementary_Data

## Supplementary data

Figure S1 and Tables S1 to S5 are available as Supplementary data at *JAC-AMR* Online.
