# Supplementary material for: Bloodstream infections after solid organ transplantation: clinical epidemiology and antimicrobial resistance (2016–21)
Source: JAC Antimicrob Resist. 2024 Jan 11;6(1):dlad158. doi: 10.1093/jacamr/dlad158 (PMC10783261; doi:10.1093/jacamr/dlad158)
Supplement: dlad158_Supplementary_Data [file dlad158_supplementary_data.docx]

**Journal of Antimicrobial Chemotherapy – Antimicrobial Resistance**

**Title:** Bloodstream Infections After Solid Organ Transplantation: Clinical Epidemiology and Antimicrobial Resistance (2016-2021)

**Authors:** Max W. Adelman, Ashton A. Connor, Enshuo Hsu, Ashish Saharia, Constance M. Mobley, David W. Victor, III, Mark J. Hobeika, Jiejian Lin, Kevin A. Grimes, Elizabeth Ramos, Claudia Pedroza, Elizabeth W. Brombosz, R. Mark Ghobrial, Cesar A. Arias

**SUPPLEMENTARY MATERIAL**

**Table S1**. Resistance profile definitions according to organism.

| **Organism type** | **Resistance Profile** | **Definition** |
| --- | --- | --- |
| *Staphylococcus aureus* | MRSA | Resistant to oxacillin or cefazolin |
| *Enterococcus* species | VRE | Resistant to vancomycin |
| Enterobacterales | ESBL | Intermediate or resistant to at least one of cefepime, ceftriaxone, cefotaxime, ceftazidime, ceftolozane/tazobactam |
|  | CRE | Resistant to at least one of imipenem, meropenem, ertapenem, or doripenem |
| *Pseudomonas aeruginosa* | CNSPA | Intermediate or resistant to at least one of imipenem, meropenem, ertapenem, or doripenem |
| *Candida* species | ARC | Resistant to at least one of fluconazole, posaconazole, voriconazole, itraconazole, or isavuconazole |

Abbreviations: ARC, azole-resistant *Candida*; CNSPA, carbapenem non-susceptible *Pseudomonas aeruginosa*; CRE, carbapenem-resistant Enterobacterales; MRSA, methicillin-resistant *Staphylococcus aureus*.

**Table S2.** Typical institutional goal maintenance immunosuppression regimen by organ transplant type for the first year after organ transplantation.

| **Medication** | **Kidney** | **Liver** | **Heart** |
| --- | --- | --- | --- |
| Mycophenolate mofetil | 1500 mg every 12 hours | 1000 mg every 12 hours | 1500 mg every 12 hours |
| Prednisone | 5 mg daily | None | 5 mg daily |
| Tacrolimus | Target level 6-8 ng/ml | Target level 3-12 ng/ml depending on patient characteristics | Target level 8-12 ng/ml |

**Table S3.** Typical institutional antimicrobial prophylaxis regimen by organ transplant type for the first year after organ transplantation.

| **Medication** | **Kidney** | **Liver** | **Heart** |
| --- | --- | --- | --- |
| Trimethoprim-sulfamethoxazole | 400 mg/80 mg daily x 1 year | 800 mg/160 mg MWF x 1 year | 800 mg/160 mg MWF x 1 year |
| Fluconazole | Not routinely indicated | 400 mg daily if 22 ≤ MELD ≤ 29 or patient hospitalized (non-ICU) for > 48 hours prior to transplant; for 42 days post-transplant | Not routinely indicated |
| Voriconazole | Not routinely indicated | 200 mg twice daily if MELD ≥ 30 or patient in ICU at time of transplant; for 90 days post-transplant | 200 mg twice daily for 90 days post-transplant |
| Other antibiotics | Standard surgical prophylaxis | Standard surgical prophylaxis | Ceftriaxone 2 g IV daily and vancomycin 15 mg/kg IV every 12 hours x 2 days each |

Only medications with activity against organisms of interest in this analysis (bacteria and *Candida* spp.) are listed. Abbreviations: IV, intravenous; MELD, Model for End-Stage Liver Disease score; MWF, Monday/Wednesday/Friday.

**Table S4**. Organisms causing BSIs in the year after solid organ transplantation.

|  | **Solid Organ Transplant Type** | | | | |
| --- | --- | --- | --- | --- | --- |
| **Organism** | **Overall (N=323)** | **Kidney (N=87)** | **Liver (N=133)** | **Heart (N=26)** | **Multi-organ (N=77)** |
| *Klebsiella* spp*.* | 68 (21.1%) | 20 (23.0%) | 25 (18.8%) | 4 (15.4%) | 19 (24.7%) |
| *Escherichia coli* | 64 (19.8%) | 30 (34.5%) | 15 (11.3%) | 4 (15.4%) | 15 (19.5%) |
| *Enterococcus faecium* | 51 (15.8%) | 2 (2.3%) | 34 (25.6%) | 3 (11.5%) | 12 (15.6%) |
| *Pseudomonas aeruginosa* | 27 (8.4%) | 7 (8.0%) | 13 (9.8%) | 5 (19.2%) | 2 (2.6%) |
| *Staphylococcus aureus* | 21 (6.5%) | 11 (12.6%) | 6 (4.5%) | 1 (3.8%) | 3 (3.9%) |
| Coag-neg. *Staphylococcus* spp.^a^ | 19 (5.9%) | 5 (5.7%) | 7 (5.3%) | 1 (3.8%) | 6 (7.8%) |
| *Enterococcus faecalis* | 12 (3.7%) | 1 (1.1%) | 5 (3.8%) | 4 (15.4%) | 2 (2.6%) |
| *Candida glabrata* | 8 (2.5%) | 3 (3.4%) | 3 (2.3%) | 0 (0%) | 2 (2.6%) |
| *Enterobacter* spp*.* | 8 (2.5%) | 1 (1.1%) | 2 (1.5%) | 1 (3.8%) | 4 (5.2%) |
| *Candida parapsilosis* | 7 (2.2%) | 0 (0%) | 3 (2.3%) | 0 (0%) | 4 (5.2%) |
| Anaerobes^b^ | 5 (1.5%) | 0 (0%) | 4 (3.0%) | 0 (0%) | 1 (1.3%) |
| *Citrobacter* spp. | 5 (1.5%) | 1 (1.1%) | 1 (0.8%) | 2 (7.7%) | 1 (1.3%) |
| *Raoultella* spp*.* | 5 (1.5%) | 1 (1.1%) | 2 (1.5%) | 1 (3.8%) | 1 (1.3%) |
| *Achromobacter* spp*.* | 4 (1.2%) | 1 (1.1%) | 2 (1.5%) | 0 (0%) | 1 (1.3%) |
| *Cutibacterium acnes* | 4 (1.2%) | 2 (2.3%) | 1 (0.8%) | 0 (0%) | 1 (1.3%) |
| Other *Enterococcus* spp*.* | 4 (1.2%) | 0 (0%) | 4 (3.0%) | 0 (0%) | 0 (0%) |
| *Serratia* spp*.* | 4 (1.2%) | 0 (0%) | 2 (1.5%) | 0 (0%) | 2 (2.6%) |
| Other gram positives^c^ | 2 (0.6%) | 1 (1.1%) | 1 (0.8%) | 0 (0%) | 0 (0%) |
| *Streptococcus* spp. | 2 (0.6%) | 1 (1.1%) | 1 (0.8%) | 0 (0%) | 0 (0%) |
| *Candida dublinensis* | 1 (0.3%) | 0 (0%) | 0 (0%) | 0 (0%) | 1 (1.3%) |
| *Proteus* spp*.* | 1 (0.3%) | 0 (0%) | 1 (0.8%) | 0 (0%) | 0 (0%) |
| *Stenotrophomonas maltophilia* | 1 (0.3%) | 0 (0%) | 1 (0.8%) | 0 (0%) | 0 (0%) |

^a^Coagulase-negative *Staphylococcus* species were included only if they were not considered a contaminant (i.e., two isolates within 3 calendar days of each other) per CDC guidelines [19].

^b^Included *Clostridium sordellii*, *Prevotella melaninogenica*, *Veillonella parvula*, anaerobic gram-negative rod (unable to further identify) and anaerobic gram-positive cocci (unable to further identify) (N=1 each).

^c^Included *Corynebacterium striatum* and *Cutibacterium avidum* (N=1 each).

Abbreviations: Coag.-neg., coagulase negative; spp., species.

**Table S5**. Factors associated with 90-day mortality among solid organ transplant patients with bloodstream infections.

| **Variable** | **Univariable OR (95% CI)** | **Multivariable OR (95% CI)**^a^ |
| --- | --- | --- |
| **Age**^a^ | 1.01 (0.97-1.06) | 1.00 (0.95-1.05) |
| **Male**^b^ | 1.38 (0.50-3.81) | 1.72 (0.55-5.34) |
| **Race**^c^ |  |  |
| Asian | 3.21 (0.31-33.00) | 2.67 (0.23-30.69) |
| Black | 1.10 (0.34-3.56) | 1.20 (0.32-4.45) |
| **CCI**^a^ | 1.05 (0.93-1.18) | 1.02 (0.95-1.05) |
| **Transplant type**^d^ |  |  |
| Liver | 2.71 (0.82-8.95) | 2.28 (0.52-10.01) |
| Heart | 2.10 (0.35-12.56) | 2.04 (0.26-15.94) |
| Multi-organ | 0.90 (0.16-5.16) | 0.77 (0.10-5.84) |
| **In ICU at transplant** | 1.03 (0.39-2.75) | 0.64 (0.20-2.14) |
| **Organism type**^e^ |  |  |
| Enterobacterales | 0.33 (0.11-0.96) | 0.42 (0.14-1.29) |
| *Enterococcus* spp. | 2.75 (1.00-7.54) | 2.17 (0.71-6.63) |
| *Pseudomonas aeruginosa* | 1.48 (0.31-7.14) | 1.07 (0.19-6.15) |
| *Staphylococcus aureus* | 0.76 (0.094-6.22) | 0.85 (0.10-7.34) |
| *Candida* spp. | 2.49 (0.49-12.66) | 3.42 (0.58-20.39) |
| Other | 0.87 (0.19-4.05) | 0.76 (0.15-3.82) |
| **MDRO type**^e,f^ |  |  |
| ESBL | 0.60 (0.13-2.74) | 0.70 (0.14-3.47) |
| VRE | 5.37 (1.77-16.31) | 5.98 (1.67-21.34) |
| MRSA | 1.91 (0.21-17.27) | 2.22 (0.21-23.18) |
| Any MDRO | 1.51 (0.59-3.92) | 1.52 (0.56-4.15) |

^a^Per one year/unit increase.

^b^Compared to female gender.

^c^Compared to white race.

^d^Compared to kidney transplant.

^e^Compared to all other bloodstream infections.

^f^There were no deaths among patients with BSIs due to azole-resistant *Candida*, carbapenem-resistant Enterobacterales, or carbapenem non-susceptible *Pseudomonas aeruginosa*, so these were not included in regression analyses.

Abbreviations: CCI, Charlson Comorbidity Index; MDRO, multi-drug resistant organism; spp., species.

**
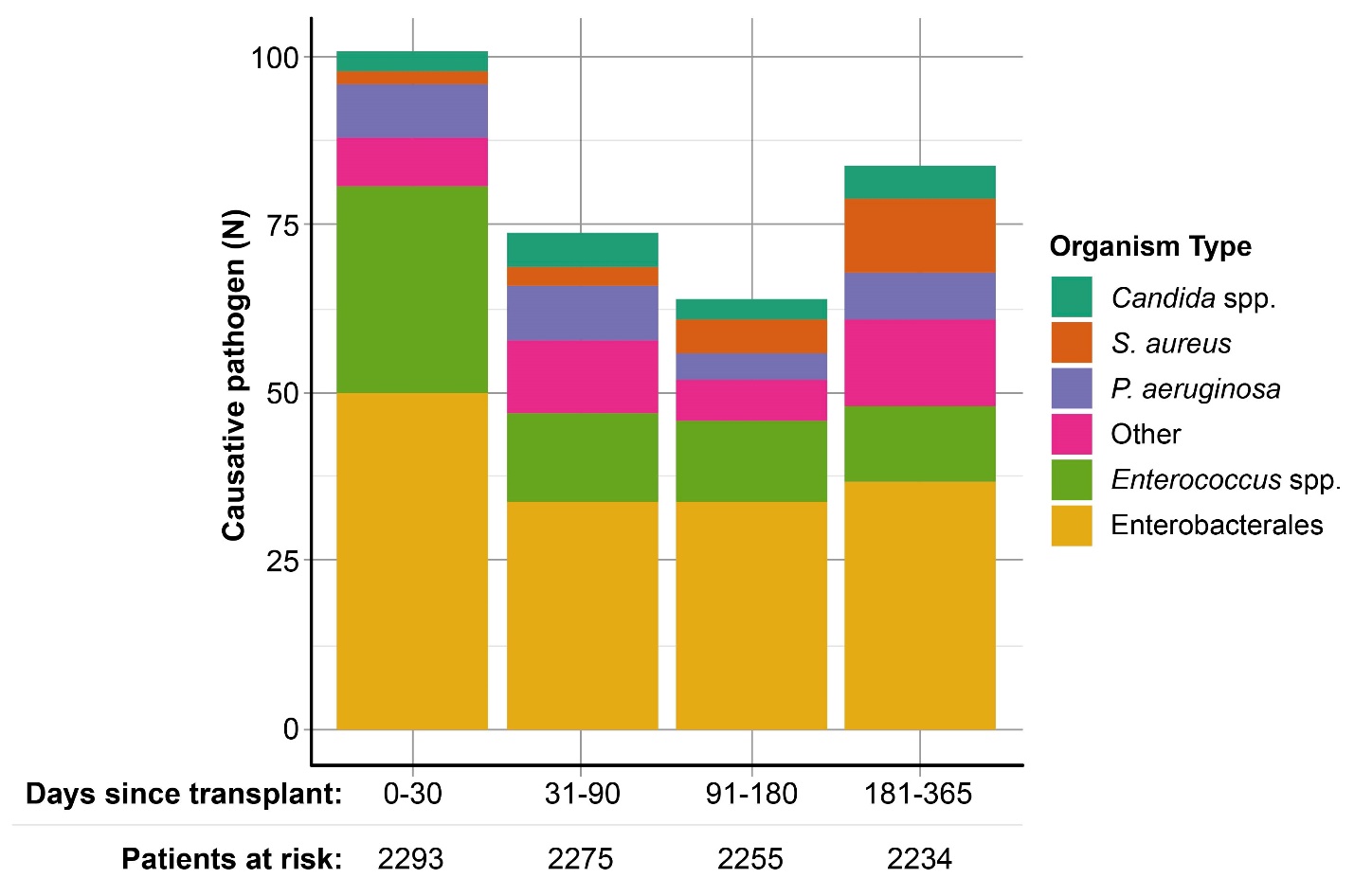
**

**Figure S1.** Organisms causing bloodstream infections after solid organ transplantation according to time from transplantation. All organ transplant types (kidney, liver, heart, multi-organ) are grouped together.
